# Using an Insect Mushroom Body Circuit to Encode Route Memory in Complex Natural Environments

**DOI:** 10.1371/journal.pcbi.1004683

**Published:** 2016-02-11

**Authors:** Paul Ardin, Fei Peng, Michael Mangan, Konstantinos Lagogiannis, Barbara Webb

**Affiliations:** 1 School of Informatics, University of Edinburgh, Edinburgh, United Kingdom; 2 Biological and Experimental Psychology, School of Biological and Chemical Sciences, Queen Mary University of London, London, United Kingdom; Northeastern University, UNITED STATES

## Abstract

Ants, like many other animals, use visual memory to follow extended routes through complex environments, but it is unknown how their small brains implement this capability. The mushroom body neuropils have been identified as a crucial memory circuit in the insect brain, but their function has mostly been explored for simple olfactory association tasks. We show that a spiking neural model of this circuit originally developed to describe fruitfly (*Drosophila melanogaster)* olfactory association, can also account for the ability of desert ants (*Cataglyphis velox)* to rapidly learn visual routes through complex natural environments. We further demonstrate that abstracting the key computational principles of this circuit, which include one-shot learning of sparse codes, enables the theoretical storage capacity of the ant mushroom body to be estimated at hundreds of independent images.

## Author Summary

We propose a model based directly on insect neuroanatomy that is able to account for the route following capabilities of ants. We show this mushroom body circuit has the potential to store a large number of images, generated in a realistic simulation of an ant traversing a route, and to distinguish previously stored images from highly similar images generated when looking in the wrong direction. It can thus control successful recapitulation of routes under ecologically valid test conditions.

## Introduction

The nature of the spatial memory that underlies navigational behaviour in insects remains a controversial issue, particularly as the neural mechanisms are largely unknown. Insects can perform path integration (PI), using a sky compass and odometer to accumulate velocity into a vector indicating the distance and direction of their start location, typically the nest or hive [1]. They are also known to be able to use landmark and/or panoramic visual memories of previously visited locations to guide their movements independently of PI [2,3]. Under normal conditions, both systems are functioning. This raises the possibility that insects additionally store PI vector information with their visual memories [4]; or link their visual memories in sequences [5] or with relative heading vectors [6], forming a topological map; or could even use the PI information to integrate their visual memories into a metric map that represents the spatial relationship of known locations [7].

However another possibility is that PI information is used to determine *which* visual memories to store, for example, the views experienced when facing the nest [8]. Subsequently, such memories can be used directly for guidance without further reference to vector information. Rotating to match the current visual experience with a stored view will give the required heading direction [9], e.g., towards the nest. Surprisingly, this navigation mechanism can exploit multiple memories without necessarily requiring recovery of the ‘correct’ memory for the current location. Baddeley et al [10,11] presented an algorithm by which an animal attempting to navigate home simultaneously compares the view experienced while it rotates to all memories ever stored while following a PI vector homewards. The direction in which the view looks ‘most familiar’, i.e., has the best match across all stored views, is generally the correct heading to take to retrace its previous path. In [11] this principle was implemented using the Infomax learning algorithm to train the weights in a two-layer network, where the input is successive images along simulated routes, and the summed activation of the output layer represents the novelty of each image. This implementation was able to replicate many features of ant route following in an agent simulation [11] and has also been shown (with some assumptions about the ant’s previous experience) to produce similar search strategies to ants in visual homing paradigms [12].

Strategies of this nature, where the animal does not need to know where it is to know where to go, have been invoked as a more parsimonious explanation for experimental results presented as evidence for a cognitive maps in insects [13]. But is this explanation of navigation plausible, given realistic environmental, perceptual and neural constraints? As pointed out in a recent review [14], parsimony based on what appears simple in computational terms may not map to simplicity with respect to the underlying neural architecture. Yet so far, “nothing is known about neural implementation of navigational space in insects” [14]. In particular, Baddeley et al [11] do not claim that the Infomax implementation of their familiarity algorithm represents the actual neural processing of the ant.

Visual processing in the ant brain has not been extensively studied, but anatomically resembles that of other insects in terms of the initial sensory layers at least. Ants have typical apposition compound eyes, but the size and resolution varies substantially across species. For the desert ants *Cataglyphis* [15] and *Melaphorus bagoti* [16] each eye subtends a large visual field (estimated at 150–170 degrees horizontal extent, with a small frontal overlap) with low visual resolution (interommatidia angles between 3 and 5 degrees). Visual signals pass through the three layers of the optic lobe (lamina, medulla and lobula) maintaining a retinotopic projection but with increasing integration across the visual field [17]. Visual signals then pass directly or indirectly to several other brain regions, including the protocerebrum, the central complex, and the calyxes of the mushroom bodies [17]. The mushroom body (MB) neuropils are conspicuous central brain structures, made up from a large number of Kenyon cells (KCs), whose dendrites together form the calyx and whose axons run in parallel through the penduculus and then bifurcate to form the vertical (or α) and medial (or β) lobes [18]. The extent of visual input to the MB is significantly greater in ants and other hymenoptera than for many other insects for which the MB input is predominantly olfactory [19]. In fact there is increasing evidence that the MB may play a role in visual learning and navigation in insects. There is evidence of expansion or reorganisation of MB at the onset of foraging in ants [20,21] and bees [22–24]. Upregulation of a learning related gene in the MB of honeybees has been linked to orientation flights in novel environments [25]. Cockroaches show impairment on a visual homing task after MB silencing [26], although the central complex rather than the MB appears essential for this task in Drosophila [27]. The MB may nevertheless play a role in some visual learning paradigms in Drosophila [28][29].

To date, the MB have been much more extensively studied in the context of olfactory associative learning, for which they appear crucial [18]. We have previously implemented a spiking neuron model of adult Drosophila MB olfactory learning [30] which used three stages of processing. Olfactory inputs produced a spatio-temporal pattern (an ‘image’ of the odour) in the antennal lobe (consistent with evidence in flies [31] but also observed in many other insects, including ants [32]). Divergent connectivity from the antennal lobe to the much larger number of KCs that make up the MB project this pattern onto a sparse encoding in a higher dimensional space [33–35]. Reward-dependent learning occurs in the synapses between the KC and a small number of output extrinsic neurons (ENs) [36–38], depending on the delivery at the synapse of an aminergic reward signal [39,40], such that each pattern becomes associated with a positive or negative outcome. This is a simplification of the insect MB circuit, which in reality includes significant feedback connectivity, synaptic adaptation at other levels including in the calyx [41,42] and has a substantial compartmentalisation of its inputs and outputs both between and within the lobes [40]. Nevertheless the basic feedforward architecture implemented in our model was shown to be sufficient to support learning of the association of non-elemental (configural) olfactory stimulus patterns to a reinforcement signal.

Our proposal here is that the MB of the ant allows it to similarly associate visual stimulus patterns, as viewed along a route, with the ‘reinforcement’ of facing, moving towards or reaching home. In fact the learnt pattern could be multimodal (e.g., including olfactory cues, see Discussion) but we focus here on demonstrating that the ecologically realistic visual learning task posed by route following could be achieved by this circuit architecture. In other words, we suggest the neural architecture of the ant MB could plausibly form the substrate for the familiarity algorithm of Baddeley et al [11]. The complexity of navigation tasks make it difficult to directly measure or manipulate neural circuits in ants under naturalistic conditions to evaluate their contribution to navigation behaviour. Instead, we take a modelling approach, and explore whether our previously developed spiking neural simulation of olfactory learning in the MB of *Drosophila* [30] could be directly applied to the complex ecological task of route memory in ants, using realistic stimuli derived directly from our field experiments.

## Results

### Reconstruction of the ant’s task

**Fig 1:**
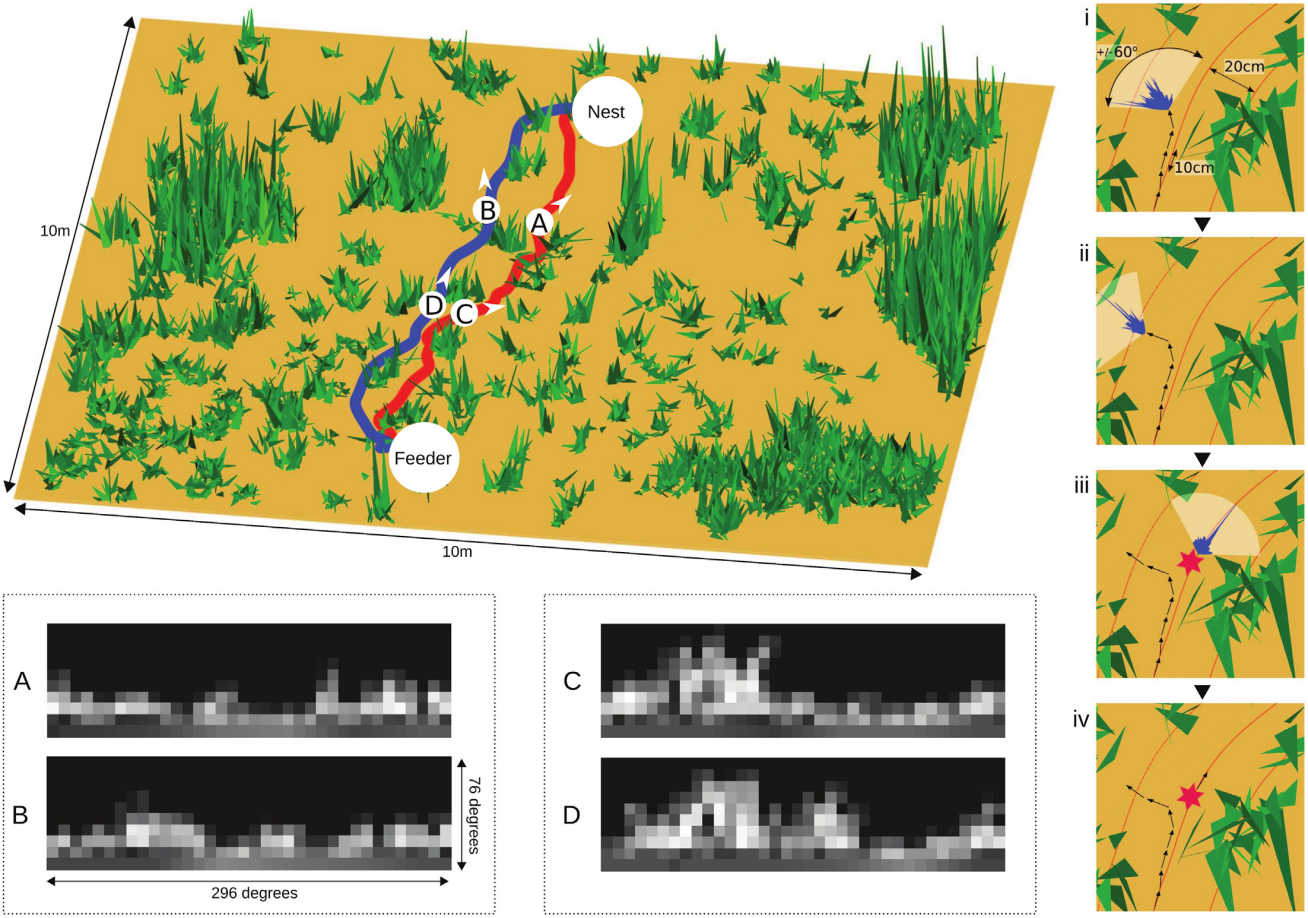
The ant’s navigational task. Left: 3D mapping of the real ant environment, which consists of flat ground and clumps of vegetation. Two actual routes followed repeatedly by individual ants from feeder to nest are shown. From a given ground point (e.g. locations A, B, C and D as indicated), the visual input of a simulated ant facing a given direction can be reconstructed, applying a 296 degree horizontal field of view, 8 degree/pixel resolution, inversion of intensity values and histogram equalization. The task of following a specific route requires distinguishing ‘familiar views’, e.g. for the red route, views A and C, from ‘unfamilar views’ e.g. B and D from the blue route, despite their substantial similarity. Right: how route following capability is assessed. The simulated ant is trained with images taken at 10cm intervals facing along a route. To retrace the route, it scans +/-60 degrees (i), evaluating the familiarity at each angle (distribution shown in blue), then moves 10 cm in the most familiar direction (ii). Deviating more than 20cm from the route is counted as an error (iii) and the ant is replaced on the nearest point of the route to continue (iv), until home is reached.

We created a realistic reconstruction of the visual experience of ants based on ecologically relevant data from our study of route following in *Cataglyphis velox* [43]. The field site was a flat semi-arid area covered in low scrub and grass tussocks. Ants were trained to forage from a feeder 7.5m from their nest. 15 individual ants were tracked over multiple trips, each revealing an idiosyncratic route to and from the feeder, which they consistently reproduced. We mapped the tussock location and size, and used panoramic pictures taken from ground level to estimate tussock height, and to generate a corresponding virtual environment, where each tussock is a collection of triangular grass blades with a distribution of shading taken randomly from the intensity range in the panoramic pictures (Fig 1). The ground is flat and featureless, and the sky is uniform, without intensity or polarized light gradients. A simulated ant can be placed at any position, with any heading, within this environment. The simulated ant’s visual input is reconstructed from a point 1cm above the ground plane, with a field of view that extends horizontally for 296 degrees and vertically for 76 degrees, with 4 degree/pixel resolution [16], producing a 19x74 pixel image. The image is inverted in intensity and histogram equalized [44] and further down-sampled to 10x36 pixels (effectively 8 degree/pixel resolution) for input to the MB network.

Fig 1 shows the potential difficulty of the route following task within this environment. The ant is surrounded by relatively dense vegetation, which blocks any distant landmarks (see also supplementary S1 Fig which shows a sequence of panoramic photographs taken from ground level along an ant’s route, illustrating the lack of any distant features visible throughout the route). The vegetation density is around 2 tussocks/m^2^ (compared to 0.05–0.75 tussocks/m^2^ used in previous simulations [11]) and ants are often observed to go directly through tussocks leading to abrupt changes in the view. As a consequence, multiple non-overlapping views need to be stored to encode the full route, and searched when recapitulating it. The views lack unique features, especially at the ant eye’s low resolution, so the potential for aliasing seems high. In particular, there is no reason to expect images seen by the ant along one route (e.g. Fig 1A and 1C) to have any common properties that could be learnt to distinguish them from non-route images (e.g. those from another ant’s route, Fig 1B and 1D; or those experienced when not aligned with the route).

### Mushroom body processing

**Fig 2:**
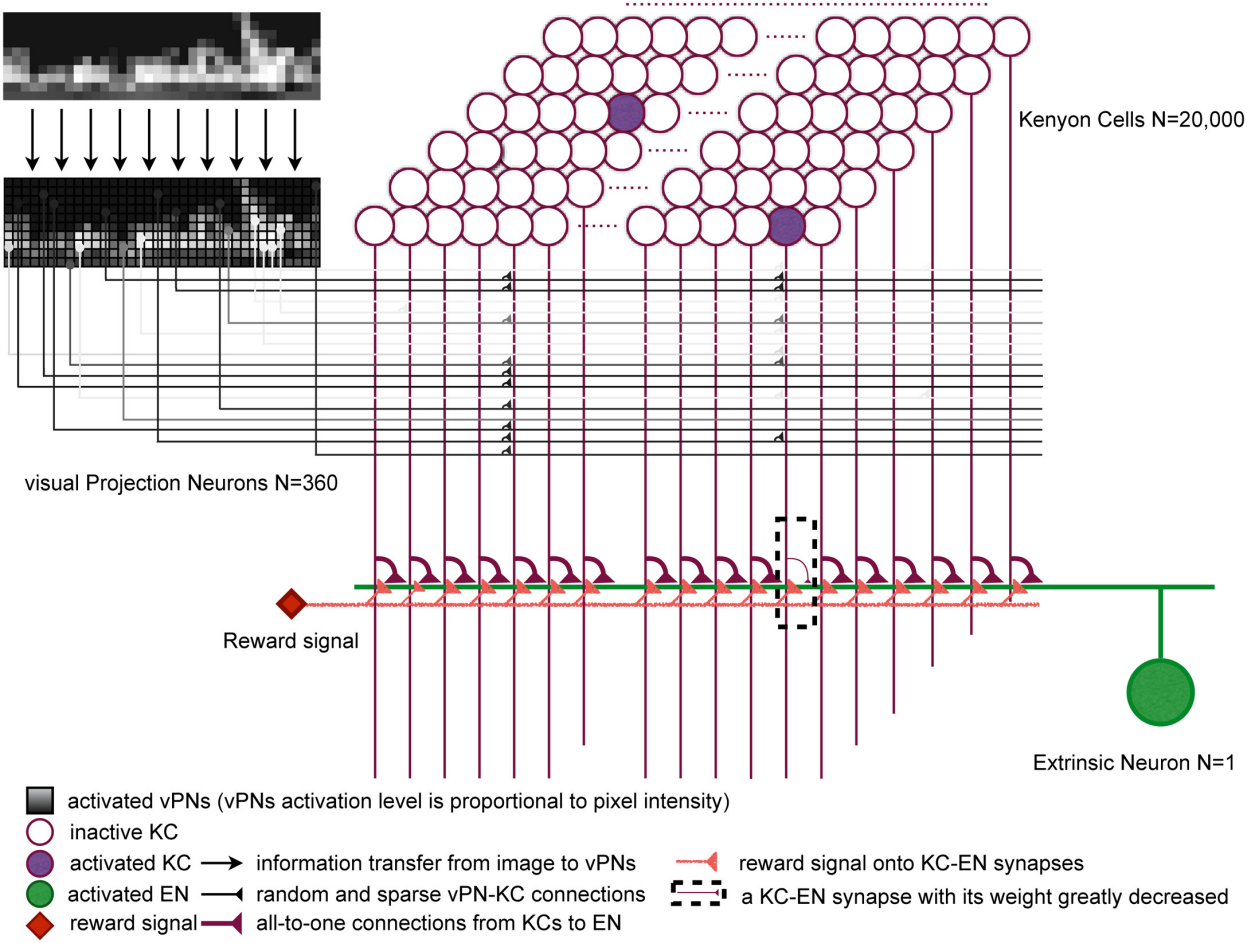
The architecture of the mushroom body (MB) model. Images (see Fig 1) activate the visual projection neurons (vPNs). Each Kenyon cell (KC) receives input from 10 (random) vPNs and exceeds firing threshold only for coincident activation from several vPNs, thus images are encoded as a sparse pattern of KC activation. All KCs converge on a single extrinsic neuron (EN) and if activation coincides with a reward signal, the connection strength is decreased. After training the EN output to previously rewarded (familiar) images is few or no spikes.

We altered our mushroom body model [30] (Fig 2) only by increasing the number of neurons (staying well below estimates for the ant brain [17]), and introducing anti-Hebbian learning [39,40]. Our input layer consists of 360 visual projection neurons (vPNs), activated proportionally to the intensity of the pixels in a scaled and normalized image (Fig 1). We intentionally kept this visual pre-processing simple, as there is little evidence on which to make assumptions about the nature of the visual input to the MB in the ant. The second layer contains 20,000—KCs, each receiving input from 10 randomly selected vPNs [35,45] (note this does not preserve retinotopy), and needing coincident activation from multiple vPNs to fire. This allows decorrelation of images using a sparse code [46], i.e., only a few KCs (around 200) will be activated, and the activation patterns will be more different than the input patterns. All KC outputs converge on a single extrinsic neuron (EN) with a three-factor rule for learning [47]. This uses the relationship of presynaptic and postsynaptic spike timing to ‘tag’ synapses, and a global reinforcement signal to permanently decrease the strength of tagged synapses, consistent with neurophysiological evidence from the MB of locusts [39]. Thus, images that have been previously paired with reinforcement will excite KCs that no longer activate EN as these connections have been weakened.

**Fig 3:**
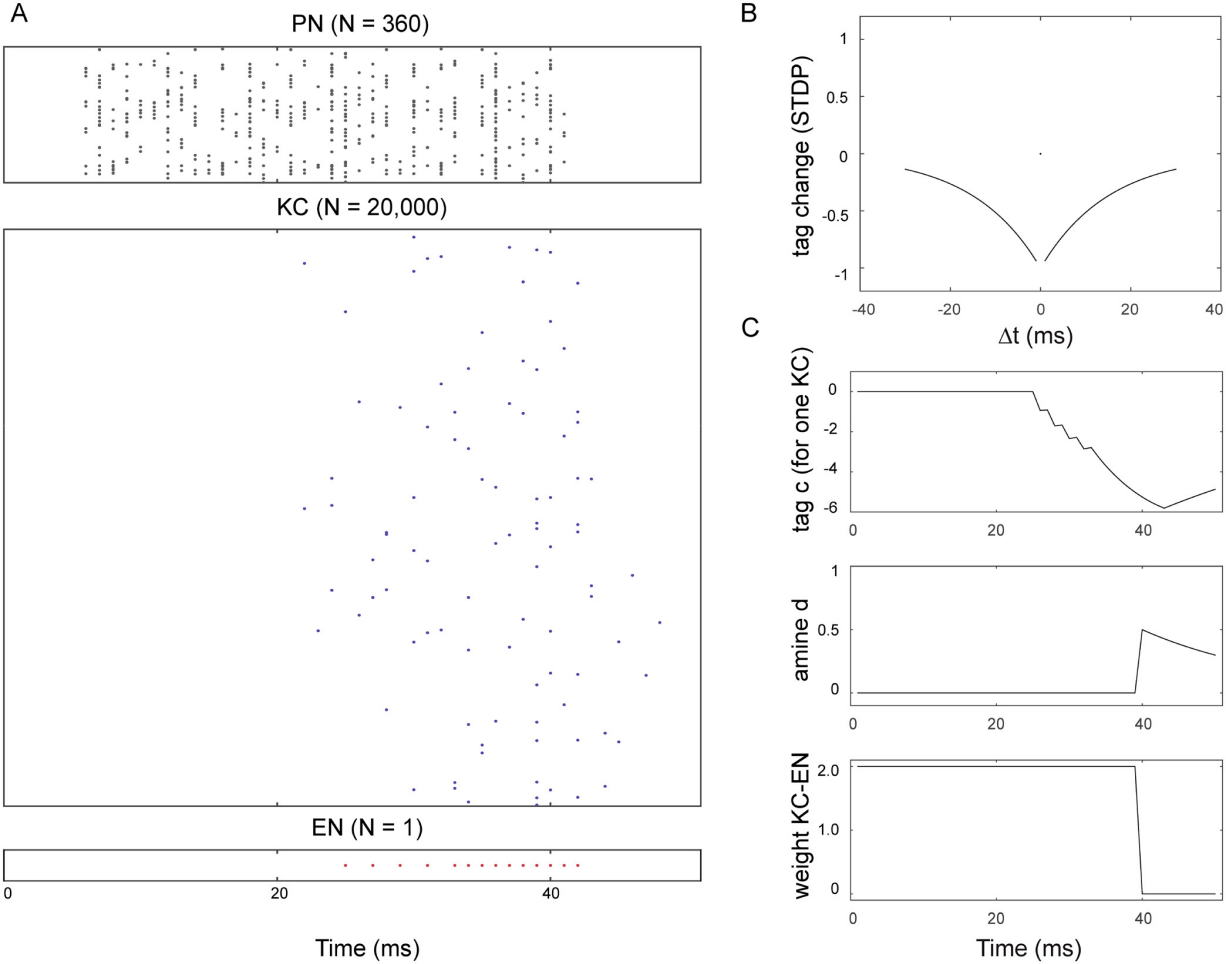
The response of the network during training with one image. A: The image is presented for 40ms, directly activating the vPNs which respond with a spiking rate proportional to the intensity of their input pixel. This produces sparse activation of the KC, which causes the EN to fire. B: An STDP process tags KC synapses depending on the relative timing, Δt, of their spikes to spikes in EN. C: Within 40ms, an active KC will have a strongly negative tag. An increase of amine d, representing reinforcement, will combine with the tag to greatly reduce the weight of the KC-EN synapse.

We assume that the ant learns a homeward route by storing the views encountered as it follows its path integration home vector back to the nest. The ‘reinforcement’ signal could thus be generated by decreases in home vector length. In practice, we train the network by generating the images that would be seen every 10cm along the ~8m recorded route of a real ant, with the heading direction towards the next 10cm waypoint. These 80 images are each presented (statically) to the network for 40ms, followed by a transient reinforcement signal (Fig 3). The parameters in the model are set to effectively result in ‘one shot’ learning given this timing of presentation: i.e., a single pairing of image and reinforcement causes the relevant synaptic weights to be reduced to near zero. Such learning is not implausible as it has been shown that, for example, individual bees can acquire odour associations in one or two trials [48]. After training, the ant should be able to recover the heading direction at any point along the route by scanning and choosing the ‘most familiar’ direction as indicated by the minima in the activity of EN during the scan.

**Fig 4:**
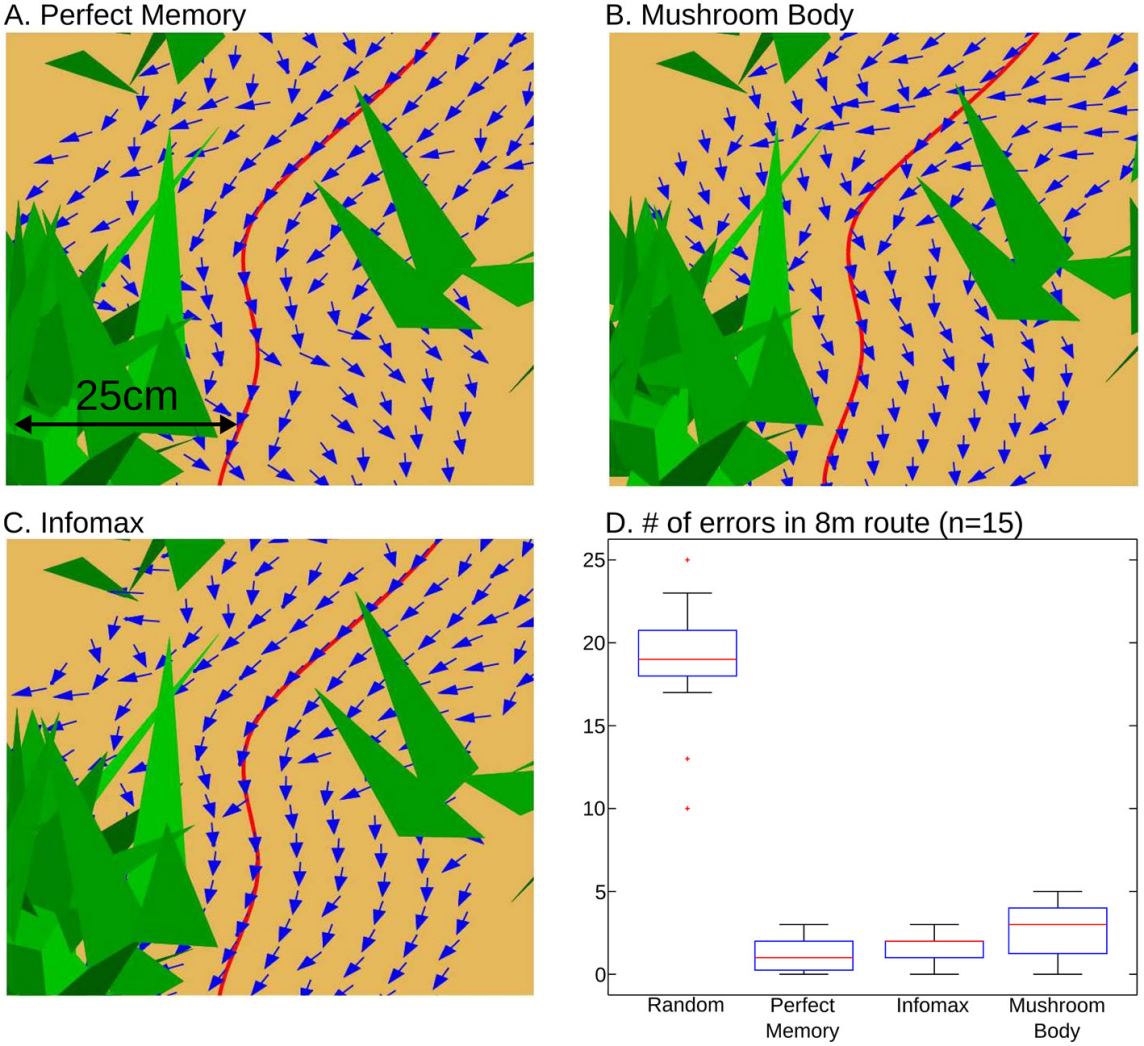
Performance of the familiarity algorithm. A-C: Perfect memory (A), the mushroom body (B) and infomax (C) are evaluated for a segment of the route (triangles are grass blades) after training with ~80 images along this route. For test locations in 5cm displacements up to 25cm away from the trained image locations (on red line), all three familiarity algorithms are robust, recovering directions (arrows) that enable route following, but even ‘perfect’ memory can produce errors when not tested at an identical location to where the image was stored. D: Comparison of number of errors made by each algorithm when retracing a route (see Fig 1), compared to random choice of direction. Boxplots show the median, interquartile range and maximum and minimum results for 15 different routes, each ~8m long, with images stored every 10cm.

We compare the MB model to two alternatives. ‘Perfect memory’ represents the best possible performance, by assuming the ant photographically stores all 80 images and directly compares the current viewpoint with all stored images to find the highest similarity, i.e., the minimum in the sum of squared pixel intensity differences (see Methods). The Infomax algorithm, used previously for this task [11] but under less realistic environmental and perceptual processing constraints, attempts to build a generative model of the 80 views using a fully connected two layer neural network (see Methods). We note that a much higher learning rate was needed in the current study to get successful results from Infomax, and it is possibly learning by overfitting, i.e., its ‘model’ consists essentially of the 80 presented views. In Fig 4A–4C we show the directional choice that would be made using the output from each method for a short segment of the path, using displacements at 5cm intervals up to +/-25cm away from the locations at which images were stored. The MB model produces directional output of equivalent reliability to the other visual memory methods, pointing the simulated ant along the route with only a few exceptions. Note that a perfect match is possible only if the simulated ant is in exactly the same location as the memory was stored, but all the methods are quite robust for nearby locations.

### Evaluating route following

We simulate retracing the route starting from the feeder, heading along the route (Fig 1, right). The next heading direction is determined by the minima in EN firing (or the equivalent choice made using Perfect Memory or Infomax) for a directional scan of ±60 degrees (reflecting a general bias to continue in the same direction). A 10cm step is taken in this direction and the process repeated until the ant reaches home. Note that it is thus possible for the simulated ant to be scanning from a slightly different position from where memory was stored, and hence for the wrong direction to be chosen, even for perfect memory. If successive movements lead the ant a significant distance from the route (> 20cm) then, in the cluttered environment we are testing, matches become poor and the ant will pursue a random course with little chance of recovery. Hence, if a step results in a location more than 20cm from the route, the error count is increased by one and the ant is placed back on the nearest point on route. We count the total number of such errors that occur before the ant comes within 20cm of the home position. As a baseline, we include a random control in which the visual information is ignored and the direction on each step is chosen randomly from ±60 degrees.

The performance of the model is assessed using 15 different ~8m routes, based on the observed routes of real ants recorded in our field study. The results are shown in Fig 4. Random directional choice produces a mean of 18.7 errors (standard deviation = 3.6) per route. Perfect memory has a much lower number of errors (mean 1.1, s.d. = 0.9) suggesting familiarity is an effective route following method. The MB model is only slightly less successful than this benchmark, with a mean of 2.6 errors (s.d. = 1.5). This is significantly worse than Perfect Memory (Bayes factor, calculated using the method in [49], is 28:1 in favour of non-equal means), but the drop in performance is small (1.5 additional errors) given the substantial reduction in computational demand − Perfect Memory requires separate comparison of the test view with every stored view for every direction in a scan, whereas MB simply uses the immediate output of the network. Infomax produces a mean of 1.5 errors (s.d. = 0.8), which is marginally better than MB (Bayes factor 2.8:1 in favour of non-equal means). But note that compared to MB, Infomax uses at least 5 times more synaptic weights, and needs to adjust every weight in the network for every training input, using a non-local rule, which is biologically less feasible. S1 Movie shows a route and images corresponding to a run in which MB produced no errors.

### Analytical solution for storage capacity of the mushroom body circuit

The key properties of the MB circuit for this task are the small number of neurons activated in the KC layer (around 1% of neurons activated by each image) and fast (one-shot) learning with no forgetting. We can abstract the spiking KCs as nodes with a binary state, representing whether or not they activated above threshold by the input pattern, and also abstract the KC-EN synaptic strength as either high (contributes to response in EN, the initial state) or low (no input to EN, the state after unidirectional, rapid learning). Because of the initially high setting (if KC is active, EN will fire), we can also simplify to a two factor learning rule: if KC activation coincides with reward, set the synapse strength permanently to low. This abstraction essentially views the learning network of the MB as a layer of binary units with outputs converging on one output unit via binary synapses that have a unidirectional plasticity rule.

Such a network is comparable to a Willshaw net [50] for which theoretical estimations of information capacity for a given number of input units N has been previously examined within a framework of fixed sparseness, binomially distributed [51]. However, the activity of each KC is not completely independent because all KCs sample from the same population of vPNs activity patterns at any given time and thus the probability distribution for the number of active KCs is not a binomial distribution [52]. Nevertheless, the expected number of active KCs is *Np*_*kc*_ (where N is the number of KCs and *p*_*kc*_ the probability of a connection between each vPN and KC) as required, while the correlation between KCs can be reduced by changing the number of vPNs sampled by each KC and thus the capacity estimation with binomial approximation may hold. Similar capacity estimations have been previously performed for networks with binary synapses and bidirectional plasticity, showing that sparseness prolongs memory lifetimes by reducing the rate of plasticity [53] and therefore the interference between new memory encoding and stored memories. Note, in our MB model, increased sparseness also influences plasticity rates and thus changes in capacity can be seen as variations in the interference of the learned patterns.

The abstracted MB allows theoretical estimation of the memory capacity *m* (the number of patterns that can be stored) of the mushroom body for a given size *N* (number of KC) and average activity *p* (average proportion of KC activated by each pattern). We derived (see Methods) an expression for the mean number of patterns *m* that can be stored before the probability of error reaches *P*_*error*_, where error is defined as having a random unlearned pattern that activates only KC nodes that already have their KC-EN weight set low, thus producing the same EN output as a learned pattern. The resulting capacity is given by:

$$m=\frac{ln\left(\frac{1-P_{error}{}^{\frac{1}{N}}}{p}\right)}{ln\left(1-p\right)}$$ (1)

**Fig 5:**
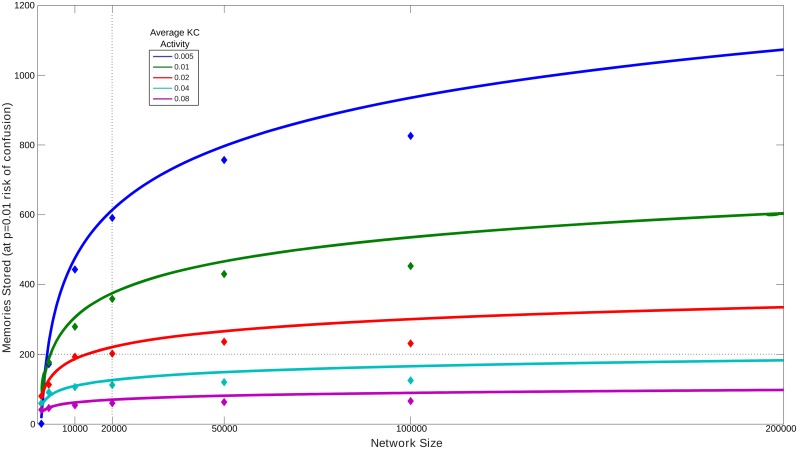
The number of independent images that an abstracted MB network can store before the probability of an error (producing an output of 0 for a novel image) exceeds 0.01. Lines: predictions from theoretical analysis. Diamonds: results from equivalent simulations using the full spiking model. The capacity scales logarithmically with the number of neurons, and increases if fewer KCs are activated on average by each pattern.

The storage capacity as a function of network size and sparsity is shown in Fig 5. Given the above assumptions, our MB model, with *N* = 20,000 input units, one output unit, and average KC activity *p* = 0.01 should allow around 375 random images to be stored before the probability of confusing a novel image with a stored image exceeds *P*_*error*_ = 0.01. We confirmed this by training our MB network with random KC patterns, and testing with 100 novel patterns. It was indeed the case that more than 350 patterns could be stored before any of the novel patterns were mistakenly classed as familiar (i.e. produced no spikes in EN, see S2 Fig). Following the same procedure for varying network size and average KC activity also produces results comparable with the theoretical predictions (Fig 5, diamonds). If memories are stored every 10cm as we have assumed, memorising 350 patterns corresponds to an ant being able to recall a route of 37.5m, or several routes of around 10m, before any confusion would occur; in uncluttered environments, memories could be more spaced (e.g. every 1m or more) and distances correspondingly increased (routes of hundreds of metres, [54]). The actual upper limit of ant route memory has not been systematically explored but these values are on the same order as those used in most experimental studies (e.g. [43,55,56]). There are also a number of plausible ways in which the capacity could be increased: e.g., having more than one EN; more states for synapses; probabilistic rather than deterministic synapse switching; or preprocessing the image data.

**Fig 6:**
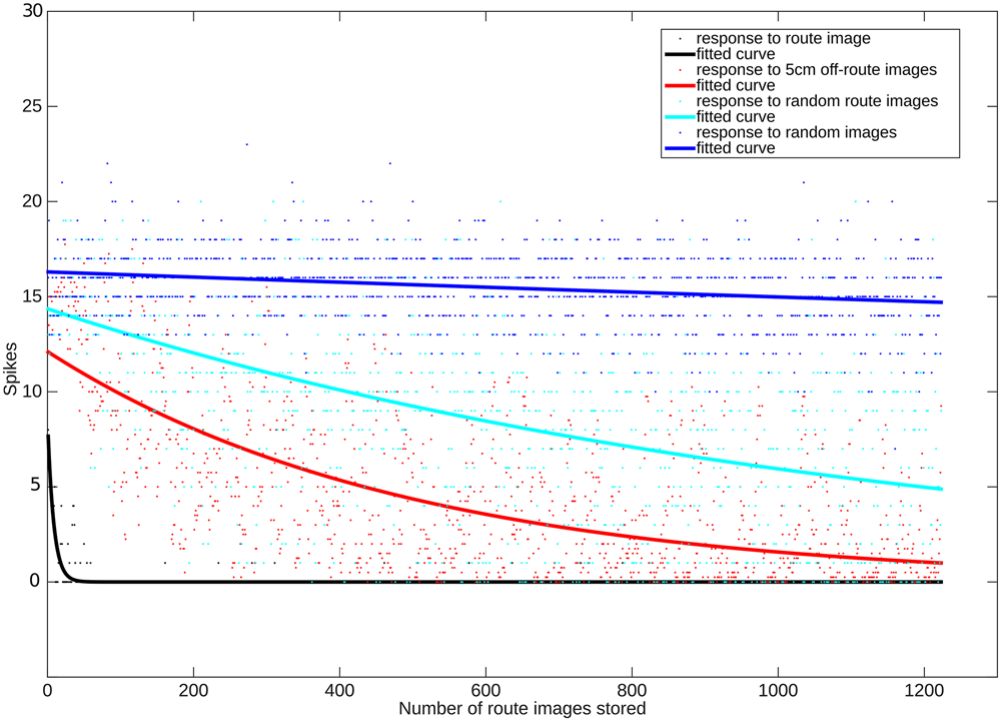
Testing the capacity of the MB network to distinguish familiar from novel images as additional route images are stored (x-axis). On average (fitted curves), the EN output to learned images is zero. Images from nearby locations with the same heading are more familiar (lower EN response) than those from random locations. Random images remain clearly distinguishable even with 1200 images stored.

To compare further the capacity estimate derived from this abstraction (with independent random input patterns) to the practical performance of the MB network (with correlated input patterns from routes) we ran the following test. The MB was trained successively with each image from every route, to a total of 1200 images. After each image was added to memory, the EN response was recorded i) for that image, ii) for an image taken 5cm away and facing the same direction, iii) for an image taken from a random location in the ant environment, and iv) for a completely random image. Distinguishing i) from ii-iv) means that stored memories are not confused with new images. Distinguishing ii) from iii) is helpful for robust route following, i.e., the right direction from small displacements should still look more familiar than random locations. Distinguishing i-iii) from iv) might be expected because completely random images will rarely look like images from the environment, where sky is always above grass, which is above the ground, etc. As shown in Fig 6, the response of EN is very noisy (plotted points), hence occasional mistakes in familiarity will occur, but nevertheless the response for stored images is on average (fitted curves) clearly distinguishable from other images as it produces no EN spikes, and images from nearby locations tend to be more familiar than images from random locations, even after storing 1200 images.

## Discussion

We have shown that the neural architecture of the insect mushroom body can implement the ‘familiarity’ algorithm [11] for ant route following. In our simulation, the MB learns over 80 on-route images, reconstructed from real ant viewpoints in their natural habitat, and reliably distinguishes them from the highly similar images obtained when looking off-route. This is the first model of visual navigation in insects to draw such a close connection to known neural circuits, rather than appealing to abstract computational capacities; as a result we found that a simpler associative learning network (computationally equivalent to a Willshaw net [50]) than Infomax suffices for the task.

Strikingly, the network which was originally developed for modelling simple olfactory associative learning in flies required almost no modification other than increased size to perform the apparently much more complex function of supporting navigation under realistic conditions. Ants (and other navigating insects such as bees) are known to have significantly enlarged MB compared to flies [57]. Visual memory has also been localised to the central complex of the insect brain [27,58], and it remains possible that navigation, including visual memory for homing routes, is carried out entirely in the central complex and does not involve the MB. We would agree that the central complex is likely to be a key area for navigational capabilities in many insects [27,59], in particular processing polarised light [60], and potentially for path integration. However the extreme memory demands of extended route following in hymenoptera such as ants and bees may have co-opted the unique circuit properties of the MB: a divergent projection of patterns into a sparse code across a very large array of neurons, enabling separation and storage of a large number of arbitrary, similar patterns. Such an architecture appears to be unique to the MB neuropils in the insect brain, and has interesting parallels to the cerebellum in vertebrates [61].

Although we have used whole images in our simulation, we believe the approach is neutral to the issue of whether insects actually use the whole panorama, the skyline, the ground pattern, optic flow, or salient landmarks. Indeed there is no reason why the patterns stored should represent only one modality at a time. The multimodal inputs to the MB in ants [62] suggest that the KC activation pattern might combine olfactory, visual and other sensory (and possibly proprioceptive or motivational) inputs into a gestalt experience of the current location, and alteration of any of these cues might reduce the familiarity. Manipulation of olfactory [63], wind [64] and tactile [65] cues have been shown to affect navigational memory in ants.

The network we have described simply stores patterns, rather than trying to learn an underlying generative model to classify ‘on-route’ vs ‘off-route’ memories. Nevertheless, in common with many learning problems, the ideal visual memory for route following has an over-fitting/generalization tradeoff. If the memory for individual views is too specific, then any small displacement from the route will result in no direction looking familiar and the animal being lost; and if the similar views resulting from small displacements could be stored as ‘one class’, the environmental range of a limited capacity memory would be increased. However, if parameters (or the learning rule) are adjusted to allow greater generalization to similar views, there will be increased aliasing and hence risk of mistaking a new view for a familiar one, which would result in moving in the wrong direction, with potentially fatal consequences. One way to deal with this problem might be to introduce more sophisticated visual processing. In particular, a more effective vPN-KC mapping than the random connectivity assumed here might be obtained by allowing self-organisation (unsupervised learning) in response to visual experience in the natural environment [66] so that only ‘useful’ correlations (for example features that vary strongly with rotation but not displacement [67]) are preserved. Evidence for adaptive synapses at this level of the circuit exists for bees [41] and flies [42]. More complex pre-processing might also be needed to deal with some aspects of the real performance of ants, who continue to follow a route under changing light conditions and movement of vegetation by wind, with some robustness to changing landmark information, and probably also pitch and roll of the head [68,69].

Similarly, we recognise there is likely to be more complexity to the ant’s use of PI and visual memory than the algorithm presented here suggests. For example, it would be necessary to postulate a separate memory to explain the outwards routes of ants, with acquisition guided by successful progress along an outbound vector that is expected to lead to a food source. Insects also appear to acquire visual memory during learning walks and flights, by turning back to view the nest [8,70,71]. In this case we would need to assume that the reinforcement signal is under more complex control of a motor programme that maintains or generates fixations in the nest direction. Another possibility is that memory storage is triggered by significant change in the view. Interestingly, this is already inherently determined by the use of a three-factor learning rule in our MB model. If a view is already sufficiently ‘familiar’, the EN will not fire in response to the KC activation, so the synapses will not be tagged and learning will not occur.

The model we present provides a neurophysiological underpinning for the claim that ants can perform route navigation without requiring a map and for assessing the range over which views can provide guidance. We should be cautious to extend results from ants to other insects, particularly bees, whose sensorimotor habitat is significantly different and might thus have developed different strategies for navigation. However, although it might seem difficult to account for novel routes (or short cuts) ever being taken using the familiarity algorithm, great care is needed to distinguish a truly novel route from the behaviour that may emerge from an animal continuously orienting to make the best visual memory match possible given its current location and stored memories. This can include visual homing within a catchment area larger than that actually explored by the animal [12,72,73]. We also note that though PI is not used in route guidance in our simulations, it will normally still be active in the animal, with potential to influence the direction taken. In particular, recent results have shown that conflicting visual and PI cues often result in a compromise direction being taken by ants [73–77]. Under some circumstances, the result could appear to be a novel shortcut.

## Methods

### Ant data

The data on ant routes and environment comes from our previous study of route following in *Cataglyphis velox* [43]. Briefly, individual foragers were tracked over repeated journeys, by marking their location on squared paper at approximately 2 second intervals relative to a grid marked on the ground with bottle caps at 1 metre spacing. Subsequently a continuous path was reconstructed using polynomial interpolation. Mapping of tussock locations and panoramic photos from this environment were used to create the virtual environment based on the same software used in [10–11]; details and the simulated world itself are available from www.insectvision.org/walking-insects/antnavigationchallenge.

### Image processing

The simulated ant’s visual input, for a specific location and heading direction, is reconstructed from a point 1cm above the ground plane, with a field of view that extends horizontally for 296 degrees and vertically for 76 degrees, with 4 degree/pixel resolution, producing a 19x74 pixel image. The greyscale image values are inverted and the local contrast is enhanced using contrast-limited adaptive histogram equalisation, by applying the Matlab function *adapthisteq* [http://uk.mathworks.com/help/images/ref/adapthisteq.html] with default values. It is then further downsampled to 10x36 pixels using the Matlab function *imresize* [http://uk.mathworks.com/help/images/ref/imresize.html] in which each output pixel is a weighted average of the pixels in the nearest 4x4 neighbourhood. For input into all models, the image is normalised by dividing each pixel value by the square root of the sum of squares of all pixel values. Matlab function *reshape*[http://uk.mathworks.com/help/matlab/ref/reshape.html] is applied to convert the normalised 2D (10x36) image into an 1D (360x1) vector used for subsequent processing.

### Spiking neural network

The Mushroom Body network is essentially the same as that described in [30] except that it uses more neurons and has anti-Hebbian learning. It is composed of three layers (see Fig 2 and Tables 1–3) of ‘Izhikevich’ spiking neurons [78], i.e., for each neuron the change in membrane potential *v*(mV) is modelled as follows:

$$C\dot{\nu }=k\left(\nu -\nu_{r}\right)\left(\nu -\nu_{t}\right)-u+I+\left[\xi \;\sim \;N\left(0,\sigma \right)\right]$$ (2)

$$\dot{u}=a\left(b\left(\nu -\nu_{r}\right)-u\right)$$ (3)

The variables *v* (membrane potential) and *u* (recovery current) are reset if the membrane potential exceeds a threshold *v*_*t*_:

$$\left\{ \begin{array}{l} \nu \leftarrow c\\ u\leftarrow u+d \end{array}\right.$$ (4)

The parameter C is the membrane capacitance, *v*_*r*_ is the resting membrane potential, *I* is the input current, *ξ* ~ *N*(0, *σ*) is noise with a Gaussian distribution, and *a*, *b*, *c*, *d* and *k* are model parameters (see Table 2) which determine the characteristic response of the neuron.

Synaptic input to the first layer, which consists of 360 visual projection neurons (vPNs), is given as an input current proportional to the corresponding pixel in the normalised image 1D vector (360x1) described above:

PN input= scaling factor×Vector

The normalization and choice of scaling factor ensures that approximately half the vPNs are activated by each image. The subsequent layers consist of 20,000 Kenyon cells (KC), each receiving input from 10 randomly selected vPNs, and a single extrinsic neuron (EN) receiving input from all KC, and their synaptic input is modelled by:

$$I=gS\left(\nu_{rev}-\nu \right)$$ (5)

where *g* (nS) is the maximal synaptic conductance (the synaptic ‘weight’), *v*_*rev*_ = 0 is the reversal potential of the synapses, and *S* is the amount of active neurotransmitter which is updated as follows:

$$\dot{S}=\;\frac{-S}{\tau_{syn}}+\phi \delta \left(t-t_{pre}\right)$$ (6)

where the parameter *ϕ* is a quantile of the amount of neurotransmitters released when a presynaptic spike occurred, *τ*_*syn*_ is the synaptic time constant, *t*_*pre*_ is the time at which the presynaptic spike occurred and *δ* is the Dirac delta function. See Table 3 for synapse parameter values. The weights from vPN to KC are fixed. The weights *g* from KC to EN are altered by learning using a modification of the three-factor rule from [79]:

$$\dot{g}=cd$$ (7)

where *g* is the synaptic conductance, *c* is a synaptic ‘tag’ which maintains an eligibility trace over short periods of time, signalling which KC was involved in activating EN (see below); and *d* is the extracellular concentration of a biogenic amine, modelled by:

$$\dot{d}=\;\frac{-d}{\tau_{d}}+BA\left(t\right)$$ (8)

where *BA(t)* is the amount of biogenic amine released at time *t*, which depends on the presence of the reinforcement signal [30] (see training procedure below) and *τ*_*d*_ is a time constant for the decay of concentration *d*.

The synaptic tag *c* is modified using spike timing dependent plasticity (STDP):

$$\dot{c}=\;\frac{-c}{\tau_{c}}+STDP\left(t_{pre}-t_{post}\right)\delta \left[\left(t-t_{pre}\right)*\left(t-t_{post}\right)\right]$$ (9)

where *δ(t)* is the Dirac delta function, *t*_*pre*_ is the time of a pre-synaptic spike, *t*_*post*_ is the time of a post-synaptic spike, and *τ*_*c*_ is a time constant for decay of the tag *c*. Thus, either pre- or post-synaptic neuronal firing will change variable c by the amount STDP, defined as:

$$STDP\left(t_{pre}-t_{post}\right)\;=\{\begin{array}{l} \;A_{+}e^{\frac{t_{pre}-t_{post}}{\tau_{+}}},\;if\;t_{pre}-t_{post}<0\\ 0,\;\;\;\;\;\;\;\;\;\;\;if\;t_{pre}-t_{post}=0\\ A_{-}e^{\frac{t_{pre}-t_{post}}{\tau_{-}}},\;\;\;\;if\;t_{pre}-t_{post}>0 \end{array}$$ (10)

*A*_+_ and *A*_−_ are the amplitudes, and *τ*_+_/*τ*_−_ are the time constants. However we use an anti-Hebbian form of this rule, such that the tag is always negative:

$$\begin{array}{l} A_{+}\;=\;A_{-}\;=\;-1.0\\ \tau_{+}\;=\;\tau_{-}\;=\;15 \end{array}$$ (11)

### Network geometry

**Table 1:** Connectivity and synaptic weights.

From X to Y	Prob. of connection	Initial Weight per connection
vPN to KC	Each KC receives input from 10 randomly selected PNs	0.25 (constant)
KC to EN	1 (all KCs connect to the EN)	2.0 (subject to learning)

**Table 2:** Parameters for each neuron type.

Parameter	PN	KC	EN
Neuron Number	360	20000	1
Scaling factor	5250	N/A	N/A
C	100	4	100
a	0.3	0.01	0.3
b	-0.2	-0.3	-0.2
c	-65	-65	-65
d	8	8	8
k	2	0.035	2
*v*_*r*_	-60	-85	-60
*v*_*t*_	-40	-25	-40
ξ	*N*(0, 0.05)	*N*(0, 0.05)	*N*(0, 0.05)

Neuron Number is the number of neurons in each type. Scaling factor is specifically for visual Projection Neurons: the input signal without scaling is the result from normalisation of grayscale images, thus in the range of [0, 1]. The scaling factor amplifies the signal so that any image will activate about half of the vPNs. The parameters from C to *v*_*t*_ in the first column are Izhikevich spiking neuronal model parameters. ξ defines Gaussian random noise current injected into each neuron.

**Table 3:** Parameters for each synapse type.

Parameter	PN to KC	KC to EN
*τ*_*syn*_	3.0	8.0
*ϕ*	0.93	8.0
g	0.25	[0, 2.0]
*τ*_*c*_	N/A	40ms
*τ*_*d*_	N/A	20ms

The parameter *τ*_*syn*_ is the time constant for synapses, *ϕ* represents the quantity of neurotransmitter released per synapse, and g is the weight per synapse. Note that the weights of connections from vPN to KC are fixed whereas the weights from KC to EN are bounded to the range of 0 to 2.0. The parameter *τ*_*c*_ is the time constant for Izhikevich type synaptic eligibility trace in KC-EN synapses, and *τ*_*d*_ is the Biogenic Amine concentration time constant.

### Training procedure

We assume that an ant learns a route while running home (following a home vector) and avoiding obstacles. This gives rise to a unique set of visual experiences which it memorises, so that subsequent traversals of the same route can be made without a home vector, and starting from any point along it. In practice, we take each recorded route of a real ant (of average length 8m) and use it to train the spiking neural network as follows. From nest to home, every 10cm along the route, we use the simulated environment to generate an image facing the next 10cm waypoint. After pre-processing as described above, the normalised image pixel values are given as input to the first layer (vPNs) of the network every 1ms for 40ms. Then a reinforcement signal is presented for one time-step (BA(t) = 0.5, for t = 40ms from image onset) and a further 10ms of simulation time (with no image or reinforcement signal presented) allowed to elapse, during which any synaptic weight changes will occur. Given the parameters below, this presentation results in rapid, ‘one-shot’ learning of the presented image, with weights between any active KC and EN decreasing rapidly to zero (see Fig 3). To save computation time, we do not explicitly model network activity during the lapse of time until the next image to be learnt would be encountered (during which the ant moves 10cm further along the route). Instead we simply reset the membrane potentials, synaptic tags and amine levels in the network to their initial state, and then present the new image for 40ms, followed by reinforcement, etc., until all the images for that route (around 80 for an 8m route) have been presented. Subsequently the spiking rate output of EN will indicate the novelty of any image presented to the network.

### Perfect memory

As a benchmark for the difficulty of the navigation task, we determine the performance of a simulated ant which has a perfect memory, i.e., it simply stores the complete set of training images, and uses direct pixel-by-pixel image differencing to compare images [9]. When recapitulating a route, novelty of the current view *I* with respect to all stored images is calculated as:

$$Novelty\left(I\right)={\text{min}}_{i}\left(\sum_{x,y}{\left(I\left(x,y\right)-V_{i}\left(x,y\right)\right)}^{2}\right)$$ (12)

Where *V*_*i*_ is each of the stored images, and *x*,*y* define the individual pixels.

### Infomax

For comparison, we also implemented the Infomax algorithm, a continuous 2-layer network, closely following the method described in [11], except with a much higher learning rate. The input layer has *N* = 360 units and the normalized intensities of the 36x10 image pixels are mapped row by row onto the input layer to provide the activation level of each unit, *x*_*i*_. The output layer also has 360 units and is fully connected to the input layer. The activation of each unit of the output layer, *h*_*i*_, is given by:

$$h_{i}=\sum_{j=1}^{N}w_{ij}x_{j}$$ (13)

Where *w*_*ij*_ is the weight of the connection from the *jth* input unit to the *ith* output unit. The output *y*_*i*_ is then given by:

$$y_{i}=\text{tanh}\left(h_{i}\right)$$ (14)

Each image is learnt in turn by adjusting all the weights (initialized with random values) by:

$$\Delta w_{ij}=\frac{\eta }{N}\left(w_{ij}-\left(y_{i}+h_{i}\right)\sum_{k=1}^{N}h_{k}w_{kj}\right)$$ (15)

Where *η* = 1.1 is the learning rate.

Subsequently the novelty of an image is given by the summed activation of the output layer:

$$d\left(\vec{x}\right)=\sum_{i=1}^{M}|h_{i}|$$ (16)

### Capacity

For this analysis we simplify the MB model by assuming the KC have a binary state (activated if input is above threshold, otherwise not active) and learning will alter the state of the respective KC-EN synapse by setting it to zero when KC activity coincides with reward. We can analyse the storage capacity of a network with *N* input nodes and average activity *p* by deriving an expression for the probability of error *P*_*error*_, defined as the probability that a random unlearned pattern activates only KC nodes that already have their weight set to 0, producing the same EN output as a learned pattern (i.e. 0). We assume the number of neurons *k* coding a pattern is drawn from the binomial distribution with probability *p*. After learning *m* random patterns:

$$P(w_{i}=0)=1-{\left(1-p\right)}^{m}$$ (17)

The probability that a new pattern activates only neurons with *w*_*i*_ = *0* is given by:

$$P_{error}=\underset{K}{\sum }\left(\begin{array}{c} N\\ k \end{array}\right){\left(p\left(1-{\left(1-p\right)}^{m}\right)\right)}^{k}{\left(1-p\right)}^{N-k}$$ (18)

That is, the sum of possible arrangements of *k* active units that all have weights set to 0. This can be written as:

$$\begin{array}{ll} P_{error} & ={\left(1-p\right)}^{N}\underset{k}{\sum }\left(\begin{array}{c} N\\ k \end{array}\right){\left(\frac{p\left(1-{\left(1-p\right)}^{m}\right)}{1-p}\right)}^{k}\\ & ={\left(1-p\right)}^{N}{\left(1+\frac{p\left(1-{\left(1-p\right)}^{m}\right)}{\left(1-p\right)}\right)}^{N}\\ & ={\left(1-p{\left(1-p\right)}^{m}\right)}^{N} \end{array}$$ (19)

For a given acceptable error rate *P*_*error*_, we can solve for the number of patterns *m* that can be stored before that error rate will be exceeded:

m= ln(1−Perror1Np)ln(1−p) (20)

Fig 5 shows how the capacity *m* of the network changes with the number of neurons *N* and average activity *p*, for *P*_*error*_ = 0.01.

To confirm this analysis is consistent with the behaviour of the full spiking network, we carried out an equivalent capacity testing process using the simulation. For a network of a given size, we generated random activation of the KC neurons at different levels of average activity. Patterns were learned as before, i.e., by applying a reward signal after 40ms, resulting in altered KC-EN weights. After each successive pattern was learned, we tested the network with 100 random patterns, to see if any produced a spiking response as low as that of the learned patterns, i.e., would potentially be confused with a learnt pattern (see S2 Fig for the result with N = 20000, p = 0.01). We noted how many patterns could be learnt before *P*_*error*_ = 0.01, i.e. where ≥ 1 out of 100 new patterns would start to be confused with a learnt pattern.

Note however this analysis and simulation assumes both learned and tested KC patterns are independent and random, which is not strictly true in the navigation task. We also tested the spiking network (with N = 20000, p = 0.01) by continuously storing additional patterns generated by real route images, and counting the spikes produced by EN for the learnt image, for an image taken from a 5cm displacement facing the same way, for an image taken at a random location in the ant environment, and for a random image. The results are shown in Fig 6.

### External database

The virtual ant world is available from: www.insectvision.org/walking-insects/antnavigationchallenge.

## Supporting Information

S1 Movie This movie shows the result of a route re-capitulation by a simulated ant using the mushroom body model, and the corresponding visual information from the ant’s point of view that is used as input to the model. The direction of each 10cm step along the route is chosen by scanning (the scanning movement is not shown) ±60 degrees and choosing the direction producing the fewest spikes from the extrinsic neuron output. The simulated ant never departs far enough from the trained route to get lost, and successfully returns home over 8 meters in the complex environment.
(ZIP)

S1 Fig A. Distant view of the study site on the outskirts of Seville, Spain with the approximate nest position indicated by the arrow. The nest is surrounded by grass shrub blocking the view of distant objects such as trees. B. Close up view of the field site with the ant nest and experimental feeder marked. C. An example route followed by an ant through the environment shown in blue from an overhead perspective. D. Panoramic images sampled along the route are shown which clearly demonstrate that distant objects were not visible to homing ants.
(EPS)

S2 Fig Capacity of a MB network with N = 20000 and p = 0.01. From Fig 5, the abstracted model provides the estimate that around 375 random images can be stored (KC weights set to 0) before the probability of an error (a new random image activates only KCs that have already had weights set to 0) exceeds 0.01. Using the full spiking network and the three factor learning rule, we train successively with 500 random KC activation patterns. After each additional pattern is stored, we test the network with 100 random patterns to see how many produce an error (have an EN output of 0 spikes, indicating a familiar pattern). More than 350 patterns could be stored before > 1/100 errors occur. The same method is used to generate data points for other values of N and p plotted in Fig 5.
(EPS)
